# Comment on Li et al. *BDP1* Variants I1264M and V1347M Significantly Associated with Clinical Outcomes of Pediatric Neuroblastoma Patients Imply a New Prognostic Biomarker: A 121-Patient Cancer Genome Study. *Diagnostics* 2021, *11*, 2364

**DOI:** 10.3390/diagnostics12030608

**Published:** 2022-02-28

**Authors:** Laura Schramm

**Affiliations:** Department of Biological Sciences, St. John’s University, New York, NY 11439, USA; schramml@stjohns.edu

In 2021, Li et al. published an article on *BDP1* variants and implications in pediatric neuroblastoma patients [1]. *BDP1* (B-double prime 1) was characterized as a subunit of TFIIIB required for accurate initiation by RNA polymerase III [2,3]. Li et al. cite Gensler’s study entitled “Negative Regulation of HER2 Signaling by the PEST-type Protein-tyrosine Phosphatase *BDP1*” as evidence for the TFIIIIB associated *BDP1* subunit as playing a key role in breast cancer [4]. In Gensler’s work, the *BDP1* protein they refer to is brain-derived phosphatase 1, as noted in Gensler’s abbreviation section [4]. Gensler et al. [4] state in the introduction of their manuscript that brain-derived phosphatase 1 (*BDP1*) belonged to the family of PEST-containing protein tyrosine phosphatases [5] and was characterized in 1996 before the human homolog of yeast *BDP1* was cloned and characterized in 2000 [2,3]. The human TFIIIB *BDP1* subunit has not been described as a PEST-containing protein tyrosine kinase. A universal nomenclature for the RNA polymerase III initiation transcription factor TFIIIB complex was adopted in 2002 [6]. The official gene symbol for brain-derived phosphatase I approved by the Hugo Gene Nomenclature Committee is PTPN18 [7]. Li et al. should review the references cited demonstrating a role for *BDP1* in human cancers [1]. The availability of genomics data makes it imperative for authors to strictly utilize the Hugo Gene Nomenclature Committee’s approved nomenclature when analyzing available datasets and citing published research studies.
